# Supplementary material for: Modulating cellular cytotoxicity and phototoxicity of fluorescent organic salts through counterion pairing
Source: Sci Rep. 2019 Oct 25;9:15288. doi: 10.1038/s41598-019-51593-z (PMC6814864; doi:10.1038/s41598-019-51593-z)

**SUPPLEMENTARY INFORMATION**

**Modulating cellular cytotoxicity and phototoxicity of fluorescent organic salts**

**through counterion pairing**

Deanna Broadwater^†^, Matthew Bates^†^, Mayank Jayaram, Margaret Young, Jianzhou He, Austin L. Raithel, Thomas W. Hamann, Wei Zhang, Babak Borhan,

Richard R. Lunt^*^, and Sophia Y. Lunt^*^

^†^Equal contribution

*Correspondence to: sophia[@msu.edu](mailto:xxxxx@xxxx.xxx) and rlunt@msu.edu

**Supplementary Figure S1. Solid state absorption coefficient versus wavelength comparison for an exemplary organic salt (CySbF_6_) and nanocrystal (PbS), both with bandgap around 1.3 eV.** The organic salt has an absorption coefficient that is orders of magnitude larger than that for the nanocrystal at wavelengths in the near-infrared around the bandgap (650-950 nm).


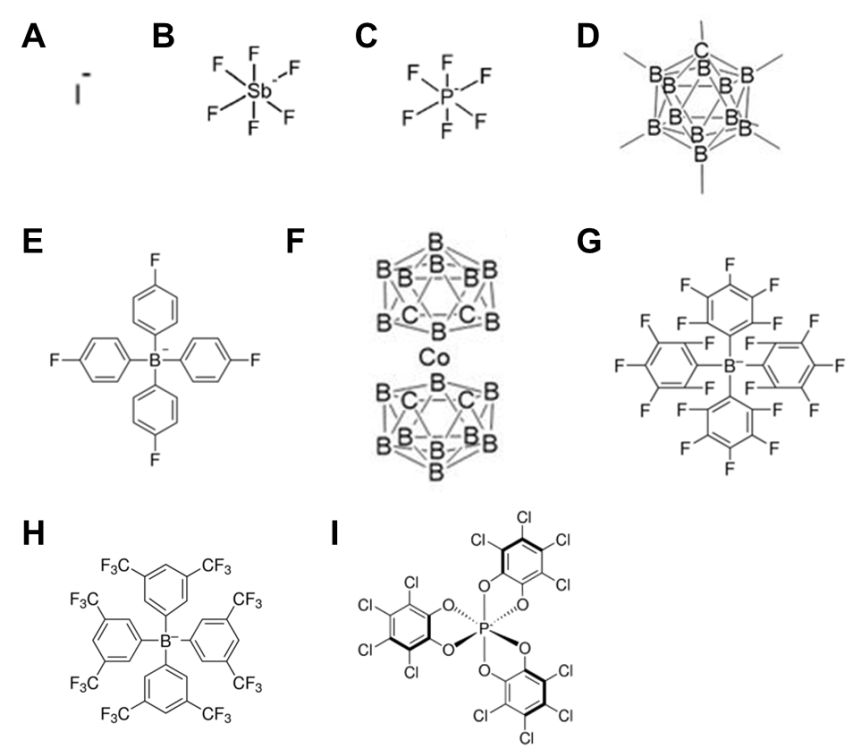


**Supplementary Figure S2. Molecular structures of anions investigated in the study.** (**A**) Iodide (I^-^) (**B**) Hexafluoroantimonate (SbF_6_^-^) (**C**) Hexafluorophosphate (PF_6_^-^) (**D**) O-carborane (CB^-^) (**E**) Tetrakis(4-fluorophenyl)borate (FPhB^-^) (**F**) Cobalticarborane (CoCB^-^) (**G**) Tetrakis (pentafluorophenyl) borate (TPFB^-^) (**H**) Tetrakis[3,5-bis(trifluoro methyl)phenyl]borate (TFM^-^) (**I**) Δ-Tris(tetrachloro-1,2-benzene diolato) phosphate(V) (TRIS^-^).


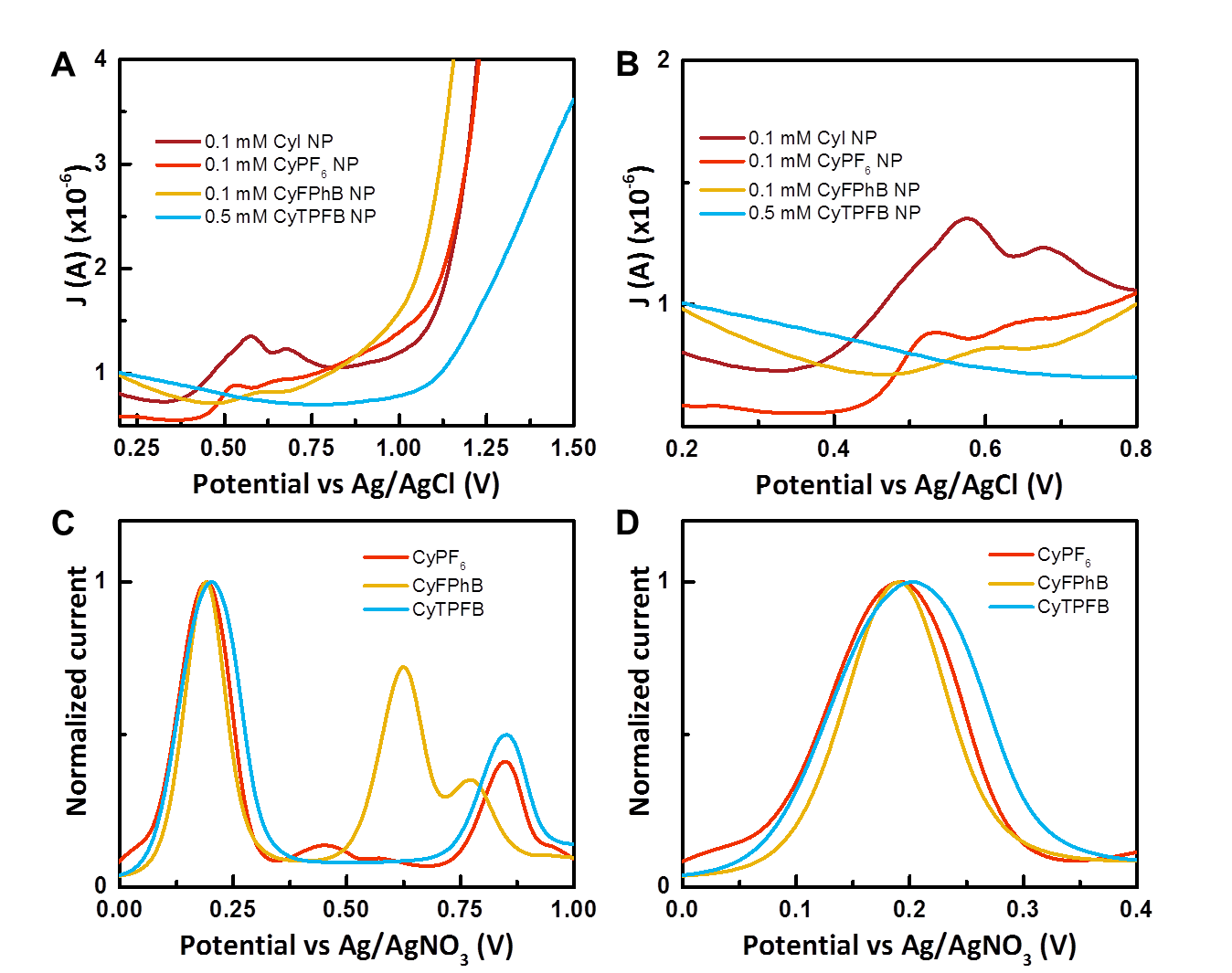


**Supplementary Figure S3. Differential pulse voltammetry measurements.** (**A**, **B**) Nanoparticles (NPs) of the salts at 0.1 mM in 10% DMSO (CyI, CyPF_6_) and 50% DMSO (CyFPhB) in water. CyTPFB NPs have greater solubility and were tested at 0.5 mM in 50% DMSO. (**C**, **D**) Monomer solutions of a representative cytotoxic, phototoxic, and nontoxic salt in acetonitrile. None of the differential pulse voltammetry measurements were performed in the presence of cells. Monomers demonstrate similar initial oxidation peaks, while nanoparticles have different peak locations. The CyTPFB nanoparticle oxidation peak is outside the redox window available for DMSO/H_2_O mixtures. A lower peak potential for CyPF_6_ compared to CyI and the shift out of the redox window for CyTPFB match anionic effects on the HOMO level shown in the solid state with UPS and correlated to redox levels. CyFPhB nanoparticles show a shift but do not fit the expected redox-HOMO trend. Monomers do not display this trend because their electronic environments are identical after dissociation due to the supporting electrolyte’s higher concentration. DMSO/water solutions were measured with a Ag/AgCl reference electrode (-45 mV vs SCE), acetonitrile with a Ag/AgNO_3_ electrode (0.36 V vs SCE).


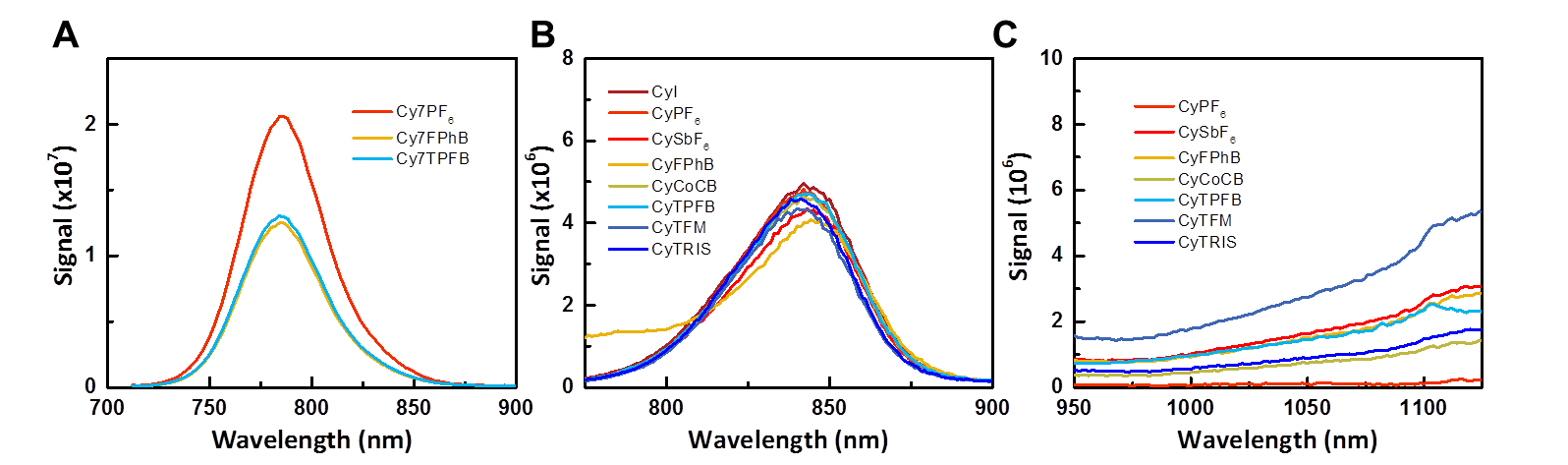


**Supplementary Figure S4**. **Photoluminescence measurements of Cy7X and CyX salts.** Absolute scale, background corrected photoluminescence spectra for (**A**) 1 μM Cy7X monomers in DMSO, (**B**) 5 μM CyX monomers in DMSO, and (**C**) 2.5 μM CyX nanoparticles in 1:99 DMSO:H_2_O. Due to the peak emission being past 950 nm, quantum yields of the nanoparticles are unmeasurable with our system, but we demonstrate here that the nanoparticles still fluoresce, albeit at different wavelengths than the monomer salts.


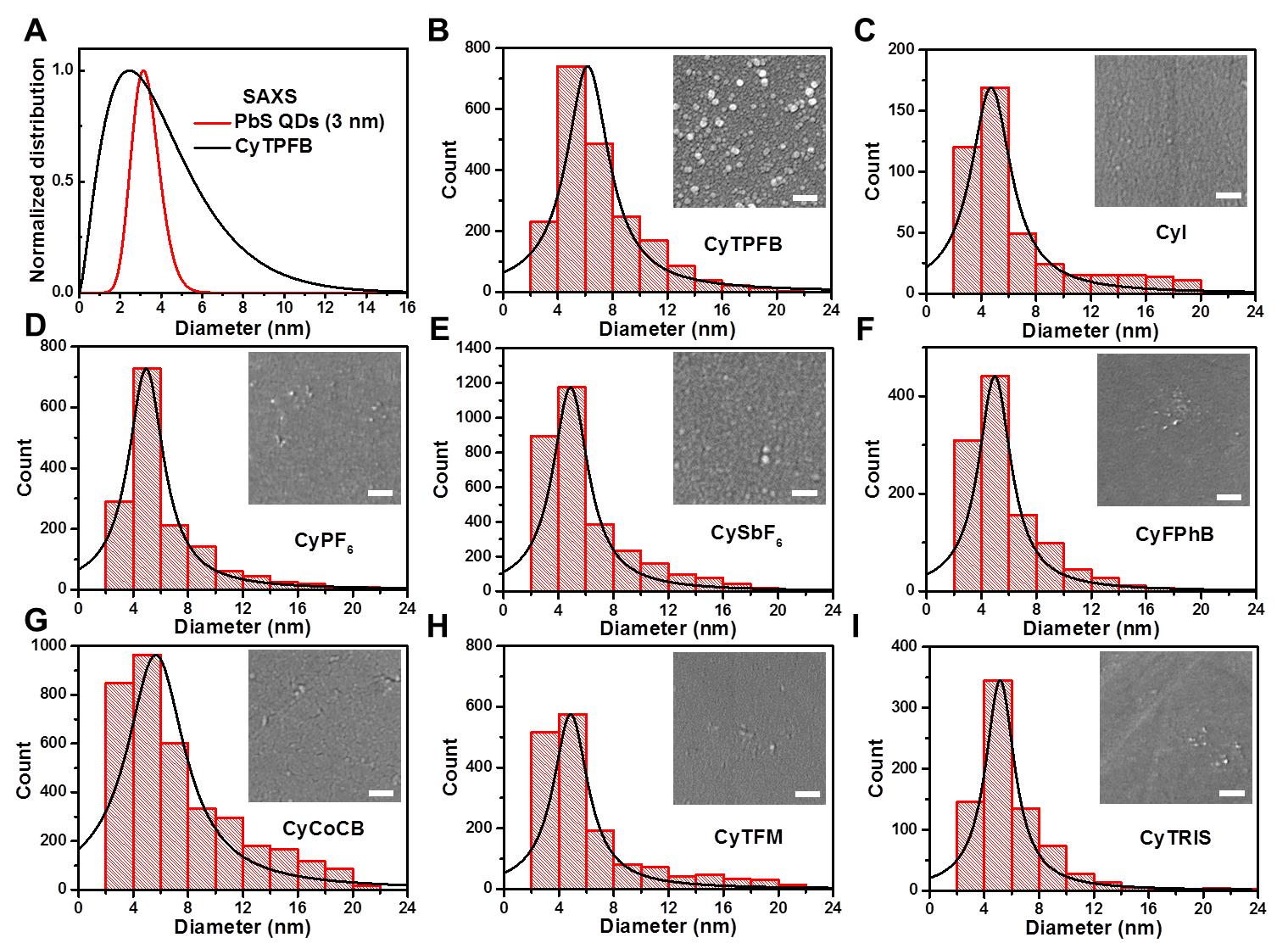


**Supplementary Figure S5.** **Nanoparticle size distribution is similar for all nanoparticles.** (**A**) Nanoparticle aggregation size distribution measurements from SAXS measurements of CyTPFB. Mean particle size is 4.1 ± 0.6 nm. PbS quantum dot size distribution is shown as a control with a nominal size of 3 nm. (**B-I**) Nanoparticle size distribution measurements from SEM images (inset, scale bar = 100 nm) of CyX. Mean aggregate size ranges from 5 to 9 nm with no observable precipitation. Other salts were examined with SAXS but did not produce usable data because of solubility limitations. SAXS requires at least 1 mg/mL of the material of interest, and such concentrations are only obtainable with CyTPFB.

**Supplementary Figure S6**. **Nanoparticle lifetime and stability is demonstrated with CyFPhB and CyTPFB.** CyPF_6_ does not form nanoparticles in cell media but nonetheless demonstrates a stable chromophore. Lifetime absorption (100-%T) data collected with UV-Vis spectroscopy for 5 µM CyPF_6_, CyFPhB, and CyTPFB in cell media. All three solutions were measured daily for 5 days and again at 8, 15, and 22 days.


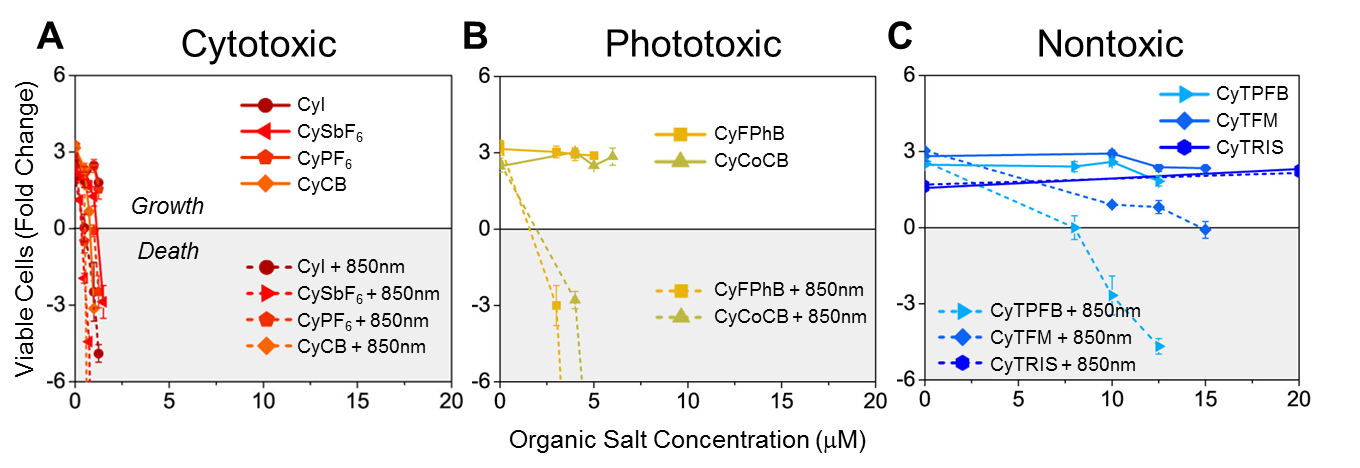


**Supplementary Figure S7. Organic salts with tunable toxicity can be used to target metastatic human melanoma cells.** Metastatic human melanoma WM1158 cells were incubated with various concentrations of Cy^+^ with different anionic pairings with or without NIR (850 nm) excitation for 4 days at which point cell viability was measured by cell count with trypan blue exclusion. (**A**) CyI, CySbF_6_, CyPF_6_, and CyCB (red/orange) are cytotoxic at low concentrations (1μM), with and without NIR excitation (cyototoxic). (**B**) CyFPhB and CyCoCB (yellow/green) do not display significant toxicity without light activation, but when photoexcited they induce significant cell death (phototoxic). (**C**) CyTPFB, CyTFM, and CyTRIS (blue) display low toxicity with and without light (nontoxic). This data agrees with the trend observed in A549 cell toxicity. Data are displayed as means ± S.E.M., *n* = 3.


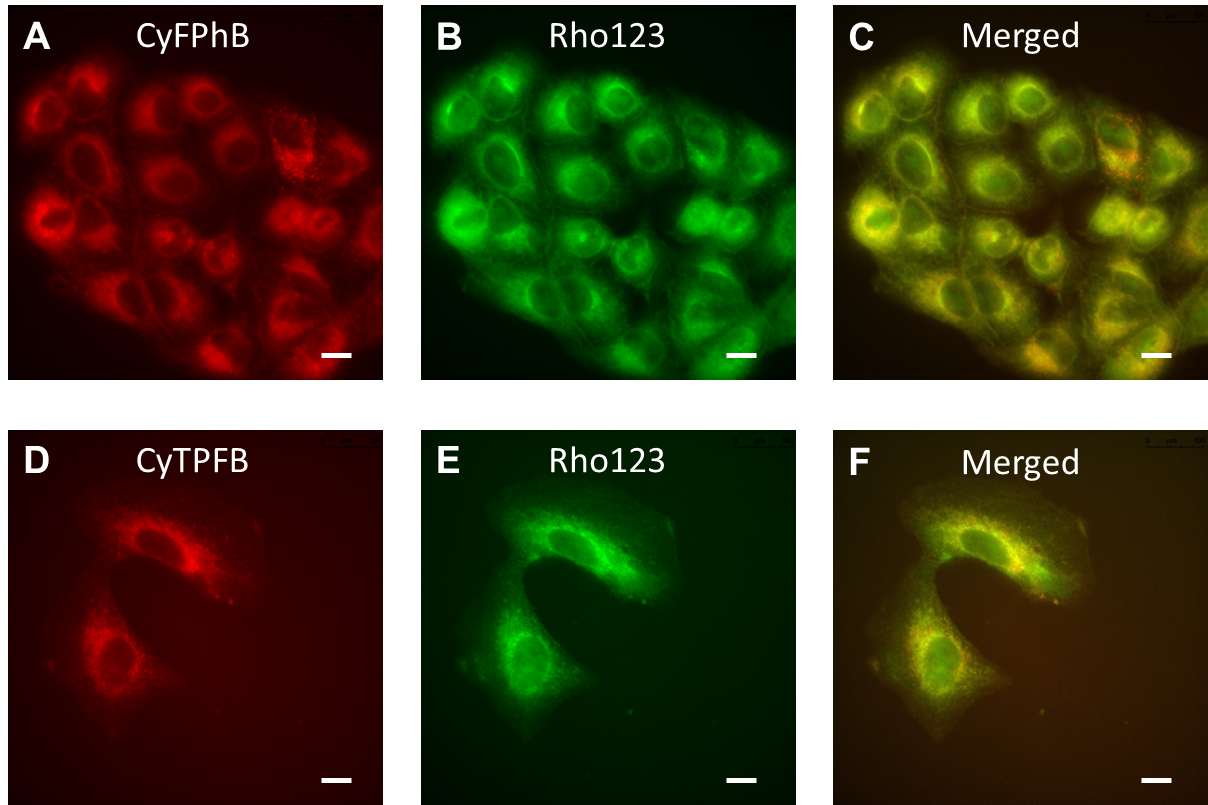


**Supplementary Figure S8.** **Fluorescent organic salts preferentially accumulate in the mitochondria and lysosomes of cells.** A549 cells were treated with either 5 μM CyFPhB or 15 μM CyTPFB. (**A**) CyFPhB staining. (**B**) Mitochondrial staining using Rhodamine 123 (Rho123). (**C**) Superimposed CyFPhB + Rho123 staining. (**D**) CyTPFB staining. (**E**) Mitochondrial staining using Rho123. (**F**) Superimposed CyTPFB + Rho123 staining. Scale bar = 20 µm (40x).


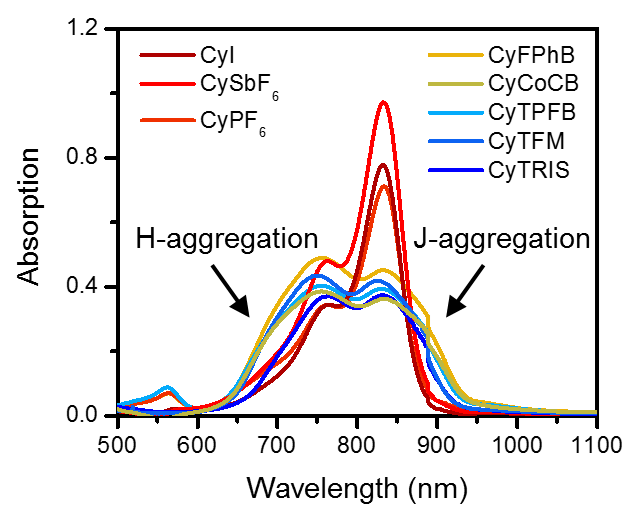


**Supplementary Figure S9. Different ion pairings display varying degrees of nanoparticle stability in cell media.** Organic salts fully dissolved in DMSO have a clear maximum at 830 nm with a leading shoulder when characterized with UV-Vis spectroscopy. After nanoparticle formation and introduction into cell media, combinations of H- and J-aggregation of organic salts can still be seen by blue-shifted peaks (lower wavelength) and red-shifted peaks (higher wavelength), respectively. This is not observed in smaller anions (I^-^, SBF_6_^-^, PF_6_^-^), indicating a lack of stability in maintaining nanoparticle formation.


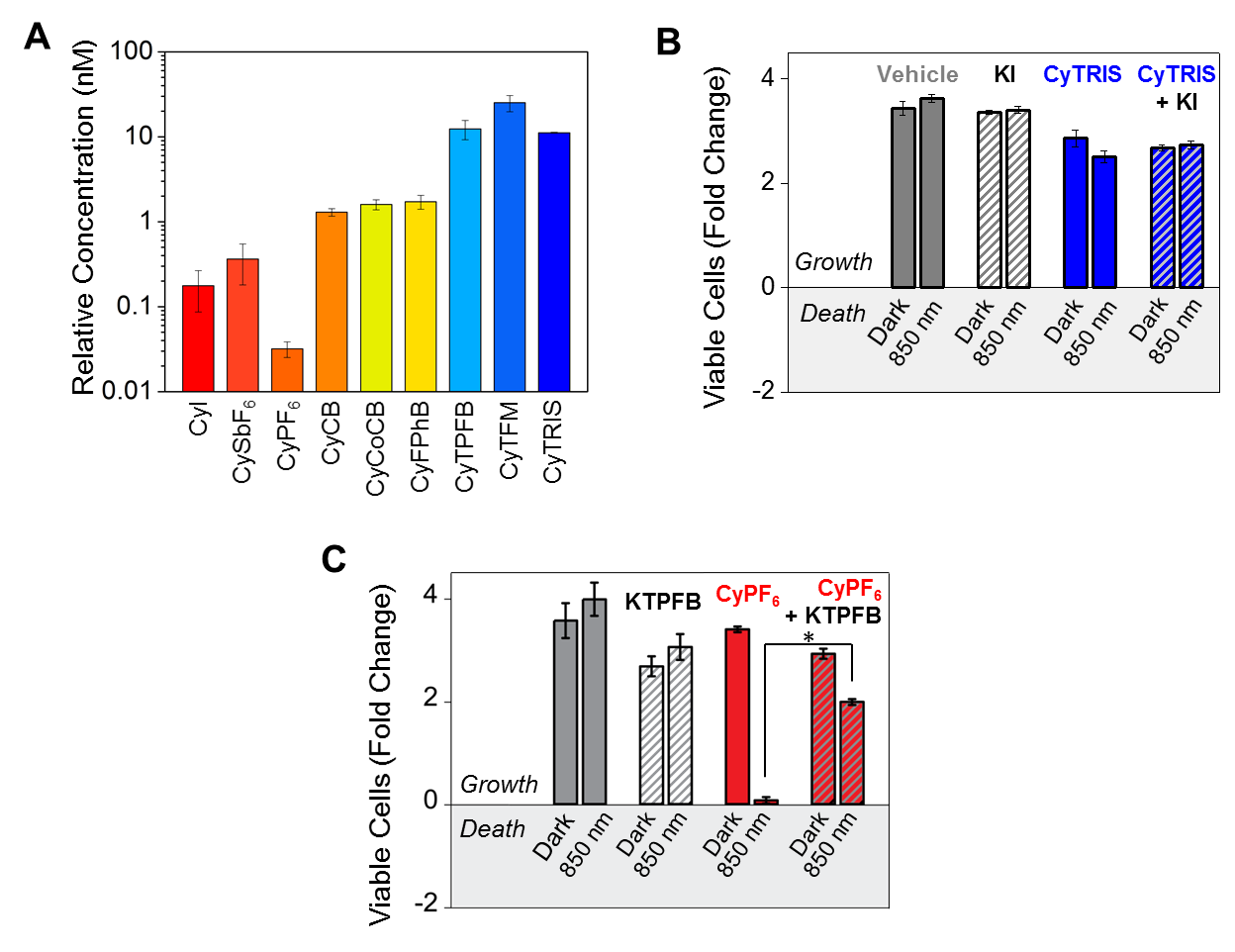


**Supplementary Figure S10. Tunability in phototoxicity is not due to cellular accumulation or counterion toxicity.** (**A**) Intracellular organic salt accumulation by A549 cells was determined using ultra high-performance liquid chromatography-mass spectrometry. In all cases, cells were incubated with 1 μM of indicated organic salt for 30 hours. Data are displayed as means ± S.D., *n* = 3. (**B**) Iodide (I^-^) is not toxic when paired with potassium (K^+^), and KI addition does not make CyTRIS toxic. A549 cells were incubated with vehicle, 1 μM KI, 30 μM CyTRIS, or 1 μM KI + 30 μM CyTRIS with or without NIR (850 nm) excitation. Cell viability determined by trypan blue staining and cell counting. (**C**) The phototoxicity and cytotoxicity of CyPF_6_ can be mitigated by the addition of KTPFB, which is not found to be toxic. A549 cells were incubated with vehicle, 15 μM KTPFB, 1 μM CyPF_6_, or 15 μM KTPFB + 0.5 μM CyPF_6_ with or without NIR (850 nm) excitation. Data are displayed as means ± S.E.M., *n* = 3.


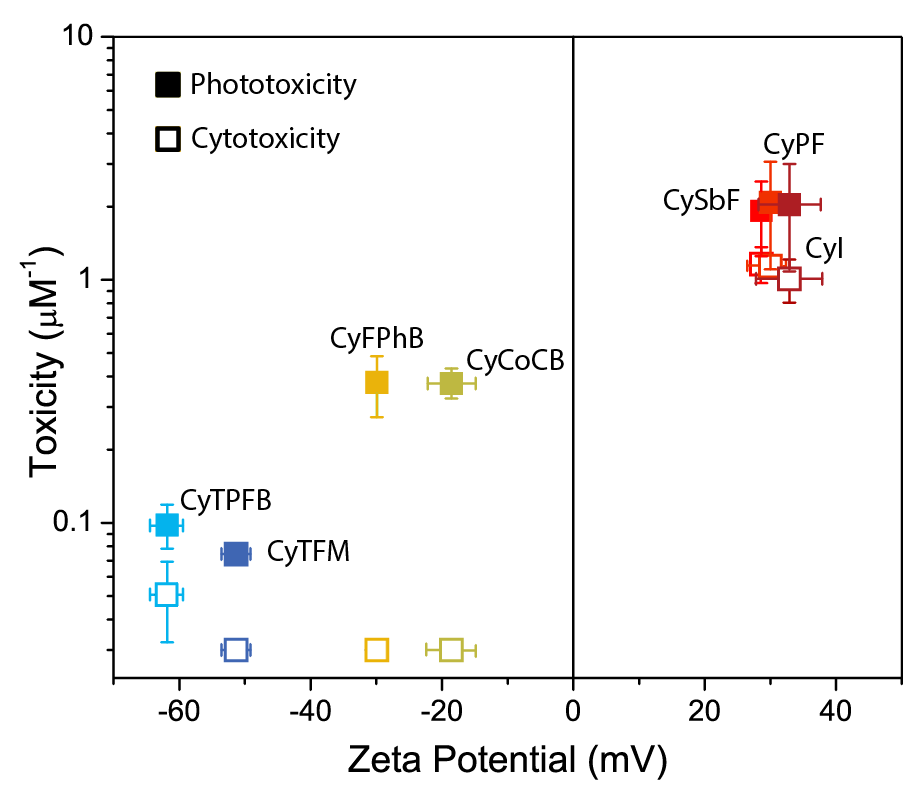


**Supplementary Figure S11. Toxicity of organic salts correlates to changes in zeta potential.** Toxicity of cyanine counterion pairings is correlated to changes in the electrostatic charge on the nanoparticles as measured by zeta potential. Cytotoxic anion pairings (red) are found to have positive zeta potentials, while non-cytotoxic pairings display negative zeta potentials. Zeta potentials were obtained from Supplementary Table S1, and toxicity values are the inverse of IC_50_ values obtained from Supplementary Table S4. Error displayed as standard deviation.


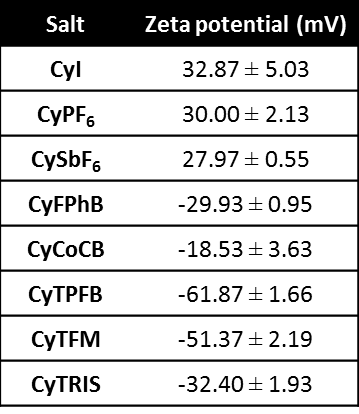
**Supplementary Table S1. Zeta potential changes as a function of counterion pairing.** Zeta potential of organic salt nanoparticles was calculated from electrophoretic mobility using a Malvern Zetasizer NS. The anion shifts the zeta potential, similar to what is observed in the solid-state using UPS (Figure 1B).


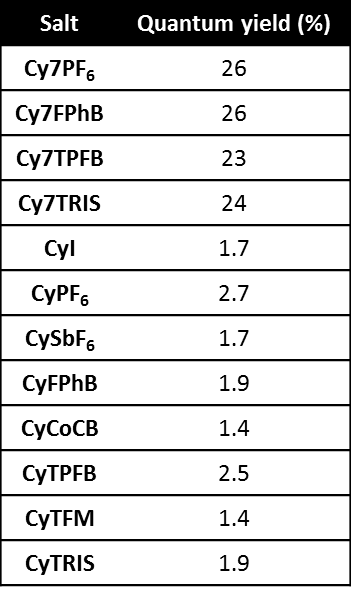
**Supplementary Table S2. Quantum yields for CyX and Cy7X salts.** The quantum yields of the monomer salts do not change significantly with the counterion. Quantum yield data for the nanoparticles is unobtainable with our system due to the emission range of the nanoparticles and the detection limits of our system in the near-infrared region.


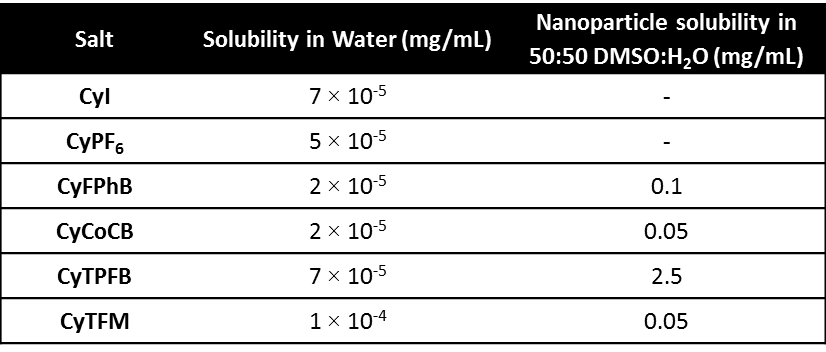
**Supplementary Table S3. CyX salts display similar solubilities in water.** The water solubility of monomer cyanines is not significantly affected by counterion pairing. SAXS measurements have been limited to CyTPFB as the only nanoparticle with high enough solubility in 50:50 DMSO:Water. Small anions such as I^-^ and PF_6_^-^ do not form nanoparticles at more than 25% DMSO.

**Supplementary Table S4. Toxicity of photoactive cation heptamethine cyanine (Cy^+^) is determined by counterion pairing.** The half maximal inhibitory concentration (IC_50_) values were generated by linear regression analysis for A549 cells. The error is displayed as a 95% confidence interval. NA implies no observable toxicity trend.

**
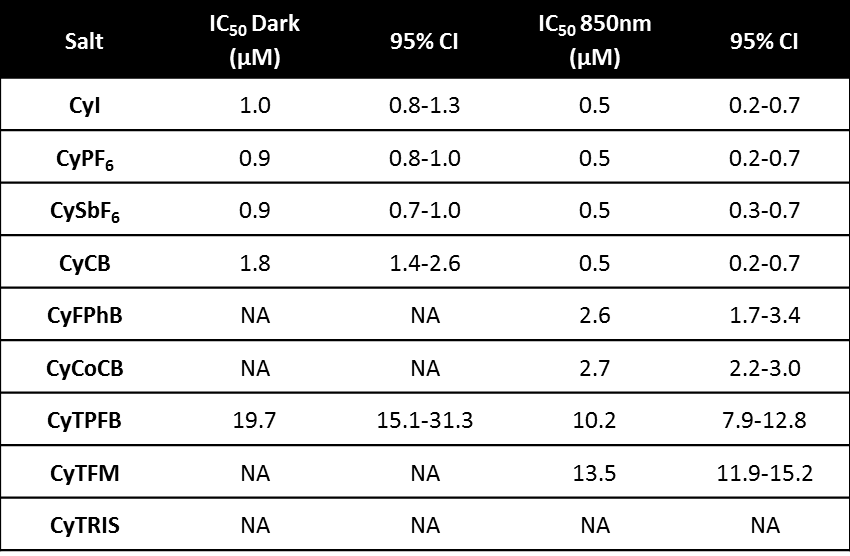
**

**Supplementary Table S5. Intracellular localization of the fluorescent ion in A549 cells does not change with the counterion.** Variables of colocalization that measure the linear relationship between red (organic salt analog) and green (Rhodamine123) fluorescence (Pearson’s coefficient), overlap of red to green area (Mander’s coefficient 1), and overlap of green to red area (Mander’s coefficient 2). All organic salts show a positive linear correlation with mitochondrial fluorescence, with similar degrees of colocalization in the mitochondria.


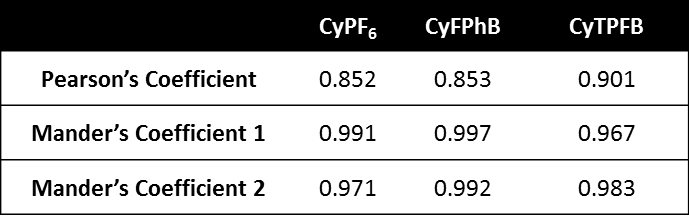

Supplement: Supplementary file 1 — Supplementary Information [file 41598_2019_51593_MOESM1_ESM.docx]
